# Similarities and divergences in the metabolism of immune cells in cancer and helminthic infections

**DOI:** 10.3389/fonc.2023.1251355

**Published:** 2023-11-16

**Authors:** Diego Esperante, Mónica Itzel Martínez Gutiérrez, Mark E. Issa, Alejandro Schcolnik-Cabrera, Fela Mendlovic

**Affiliations:** ^1^ Plan de Estudios Combinados en Medicina (PECEM), Facultad de Medicina, Universidad Nacional Autonóma de México (UNAM), Mexico City, Mexico; ^2^ Department of Neurology, Massachusetts General Hospital, Charlestown, MA, United States; ^3^ Département de Biochimie et Médicine Moléculaire, Université de Montréal, Succursale Centre-Ville, Montréal, QC, Canada; ^4^ Department of Immunology-Oncology, Maisonneuve-Rosemont Hospital Research Centre, Montréal, QC, Canada; ^5^ Departamento de Microbiología y Parasitología, Facultad de Medicina, Universidad Nacional Autónoma de México, Mexico City, Mexico; ^6^ Facultad de Ciencias de la Salud, Universidad Anáhuac México Norte, Huixquilucan, Mexico

**Keywords:** antitumoral response, helminth infection, Th1 and Th2 response, immunometabolism, immune evasion

## Abstract

Energetic and nutritional requirements play a crucial role in shaping the immune cells that infiltrate tumor and parasite infection sites. The dynamic interaction between immune cells and the microenvironment, whether in the context of tumor or helminth infection, is essential for understanding the mechanisms of immunological polarization and developing strategies to manipulate them in order to promote a functional and efficient immune response that could aid in the treatment of these conditions. In this review, we present an overview of the immune response triggered during tumorigenesis and establishment of helminth infections, highlighting the transition to chronicity in both cases. We discuss the energetic demands of immune cells under normal conditions and in the presence of tumors and helminths. Additionally, we compare the metabolic changes that occur in the tumor microenvironment and the infection site, emphasizing the alterations that are induced to redirect the immune response, thereby promoting the survival of cancer cells or helminths. This emerging discipline provides valuable insights into disease pathogenesis. We also provide examples of novel strategies to enhance immune activity by targeting metabolic pathways that shape immune phenotypes, with the aim of achieving positive outcomes in cancer and helminth infections.

## 1 Introduction

Both tumors and helminths in early stages of infection induce a Th1 response that turns into a Th2 response, in the case of helminths at very early stages of infection. In fact, helminths are among the strongest Th2 inducers in nature. Many parasite-derived molecules have been shown to have Th2-inducing properties (1). Helminth-derived products can also actively promote regulatory T cells (Tregs) and regulatory B cell (Bregs) differentiation, as well as tolerogenic dendritic cells (DCs); which are able to inhibit Th1 and Th17 responses (2, 3). This polarization from Th1 to Th2 permits long-term survival and chronic infections.

Tumors, as helminths, can subvert the immune response by different mechanisms that include modulation of the pro-inflammatory to an anti-inflammatory milieu. Tumors initially induce an M1/Th1 response that progresses to an M2/Th2 response enabling tumor growth. Immune cells, including neutrophils, macrophages, and T lymphocytes, exhibit plasticity and can polarize to pro- or anti-inflammatory phenotypes depending on the tumor microenvironment (TME) (4).

Recently a novel discipline has developed as a result of the merge between immunology and metabolism, known as immunometabolism. Outcomes of the immune response depend on the metabolic characteristics of immune and surrounding cells. Different cell phenotypes differentiate depending on the energetic and nutritional status of the microenvironment (5). Understanding the significance of the metabolic characteristics and the impact they have on cell reprogramming during cancer progression and parasitic infections holds promise to better treat and manipulate immune responses as adjuvant therapies to treat these diseases. In this review we will analyze the current knowledge of cancer immunometabolism and the incipient studies showing how helminth infections shape immune cell metabolism and function. How energetic and nutritional needs may shape responses of infiltrating immune cells in the TME or the site of helminth infection that can ultimately impact progression of the disease.

## 2 Immune mechanisms against tumors and parasites

### 2.1 Mechanisms of tumor immunosurveillance

Tumor immunosurveillance refers to the process by which innate and adaptive immunity monitor the body for the presence of malignant cells for elimination. This process occurs continuously throughout lifetime, thereby preventing initiation, development, and progression of cancer. Effector immune cells surveil tissues using a variety of mechanisms, including pathogen-associated molecular patterns (PAMPs) and damage-associated molecular patterns (DAMPs) recognition, natural direct cytotoxicity, antigen presentation, antibody-dependent cellular cytotoxicity (ADCC), and the activation of adaptive responses (6, 7).

**Figure 1:**
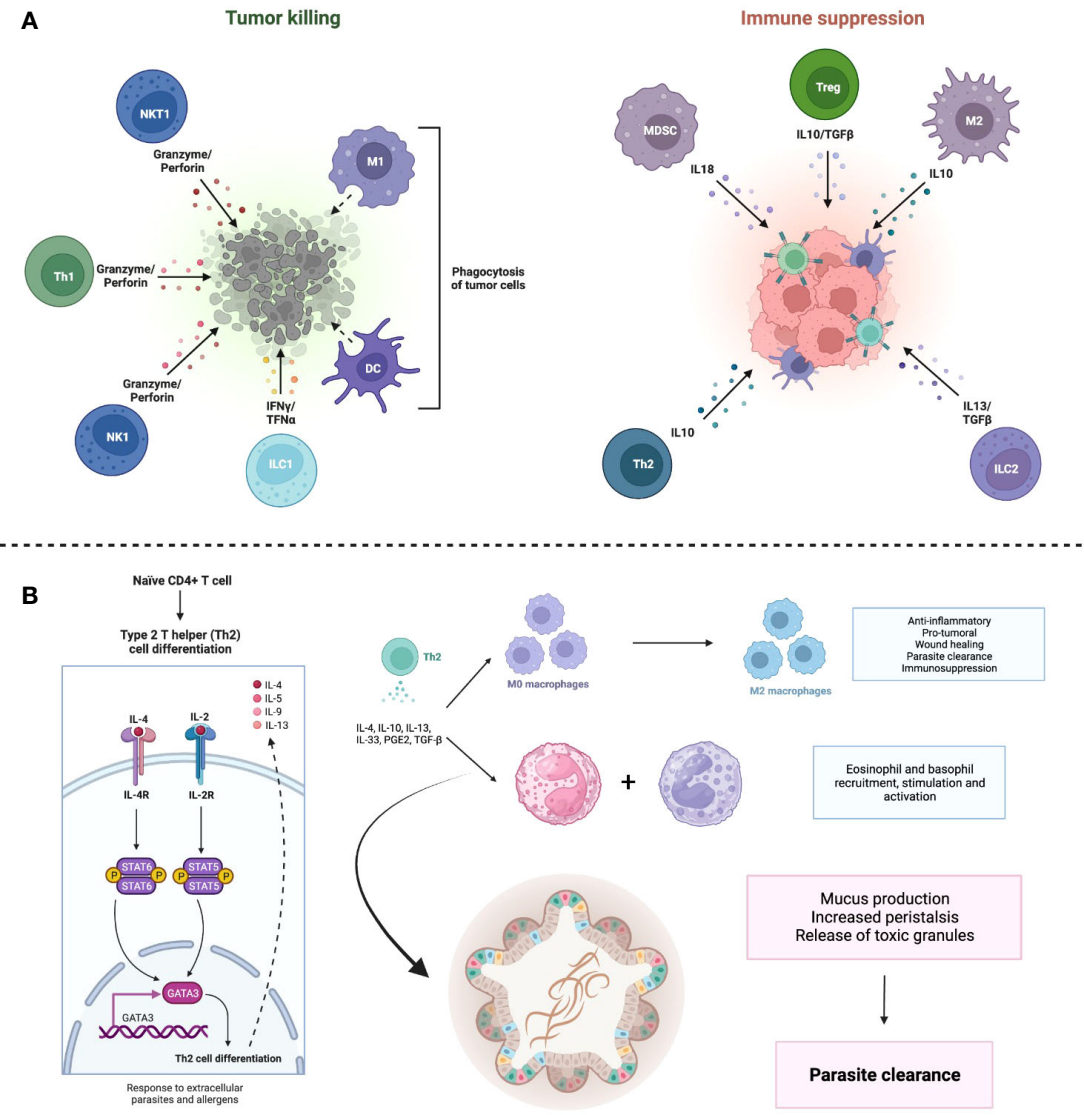
Immune response against cancer cells and helminths. **(A)** Cancer cells exhibit a mixed immune response, which can be defined as anti-tumor if it mediates cytotoxicity against cancer cells or pro-tumor if it suppresses the effector response by immune cells against the cancer cells. **(B)** Helminths elicit a Th2 response. Induction of Th2 cells requires IL-4 and IL-2; these cells, in turn, synthesize IL-4, IL-5 and IL-13, which intervene in the polarization of M2 macrophages and the recruitment of basophils and eosinophils. These cytokines also have a direct effect on the intestinal epithelium, stimulating mucus secretion and ciliary clearance of parasites (a process known as “weep and sweep”). Together with increased peristalsis, these processes result in the expulsion of parasites from the gastrointestinal tract. Created with BioRender.

Immune cells interact with tumor cells through a process known as immunoediting, which consists of three phases, namely: elimination (immunosurveillance), equilibrium, and escape (6, 7). In the elimination phase, immune cells recognize and destroy early malignant cells, thereby preventing tumor formation. This phase is mediated by various types of immune cells, including natural killer (NK) cells, DCs, macrophages, B cells and T cells (6, 7). Malignant cells eventually alter the expression of relevant ligands, thereby evading immune recognition, a state in which an equilibrium with the immune system is reached. In this phase, malignant cells survive and slowly proliferate while interacting with immune cells (6, 7). Immune cells continue to eliminate malignant cells but are unable to completely produce the desired effect, a process that is believed to last for years (8). Malignant cells ultimately acquire the necessary tools to completely evade the equilibrium phase, proliferating and possibly metastasizing to adjacent tissues (9) (**Figure 1A**).

#### 2.1.1 Natural killer cell-mediated immunosurveillance

NK cells were identified to spontaneously target tumoral cells both *in vitro* and *in vivo* without prior interactions. Mounting evidence also demonstrates that NK cells are involved in tumor rejection and allogeneic stem cell transplantation rejection in humans (10). NK cells surveil tumors and rapidly recognize and eliminate cancer cells in a tightly regulated process (11, 12). The recognition of ligands identifiable on tumoral or stressed cells and subsequent interaction stimulate activating receptors expressed on NK cell surfaces. Upon activation, NK cells initiate the secretion of inflammatory cytokines and release of cytolytic granules onto target malignant cells (13, 14).

NK cells can recognize and kill cancer cells by several mechanisms that include: i) Missing self, a mechanism where the lack of the major histocompatibility complex (MHC) class I molecules on tumor cells induces activation of NK cells; ii) Transforming cells undergo cellular stress and specialized receptors on NK cells recognize and bind to the cell surface stress-induced ligands activating cytolytic activity (15, 16); iii) Natural cytotoxicity, through “natural cytotoxicity receptors” (NCRs) that can bind to commonly expressed molecules on pathogens and damaged cells (17–19); iv) ADCC. B cells secrete antibodies specific to tumor cell neoantigens, which then engage NK cells through Fcγ receptors (aka CD16a and CD16b), activating cytolytic activity against the antibody-coated tumoral cells (20).

#### 2.1.2 Macrophages in cancer cell detection

Tumor-associated macrophages (TAMs) have been classically viewed as undesired intratumoral cells that promote tumor growth. However, a body of evidence has recently emerged and showed that TAMs exhibit two main polarities, M1 and M2. The M1 phenotype is thought to mitigate tumor growth, whereas M2 macrophages are thought to play immunosuppressive roles like those of Tregs (21).

The mechanisms underlying these effects range from nutrient depletion to Treg recruitment. A recent study has demonstrated that TAMs promote the conversion of conventional CD4+ T cells into Tregs in spontaneous models of breast cancer. The conversion was mediated by promoting CD4+ T cell PD1 expression and by TAM-derived TGF-β. Furthermore, TAM-derived IL-10 has been demonstrated to suppress the production of IL-12 from tumoral migratory inflammatory conventional DCs, thereby reducing cytotoxic CD8+ T cell activity. Arginase 1 (Arg1) is an enzyme that metabolizes L-arginine, an amino acid required for metabolic fitness, to L-ornithine and urea, rendering an M2 phenotype. Murine M2 TAMs metabolize L-arginine via Arg1 to suppress the proliferation and the inflammatory/cytotoxic activities of T cells. Arg1 inhibition mitigates tumor growth in immunocompetent mice (22–24).

#### 2.1.3 DC mechanisms of cancer cell detection

DCs play an important role in tumor immunosurveillance by constantly displaying tumoral neoantigens to T cells (25). Neoantigen-bearing DCs migrate to proximal draining lymph nodes to prime T cells activating the adaptive immune response. Conventional DCs secrete cytokines (such as tumor necrosis factor-α [TNF-α] and IL-12) that attract a variety of immune cells (such as T cells) to the lesion site and initiate a predominantly Th1 response (26, 27).

DC activation of T cells necessitates two essential molecules, CD80 and CD86. CD80 and CD86 belong to the B7 family of integral membrane proteins that function as co-stimulatory signals for T cells (28). CD80 and CD86 bind to the costimulatory receptor CD28, thereby inducing T cell proliferation and cytokine production (28). T cell activation results in the upregulation of the cytotoxic T-lymphocyte antigen-4 (CTLA-4), a CD28 homologue receptor that downregulates T cell activation (29). CTLA-4 is an inhibitory immune checkpoint that acts as an “off switch” by competing with CD28 for CD80 and CD86 to turn off T cell activation. CLTA-4 inhibition has received world attention for its role in mediating desired clinical effects in several malignancies (30).

Due to their critical role in antigen presentation and in promoting an adaptive immune response, DCs have been leveraged to engineer cancer vaccines (31). Several methods were developed to load DCs with cancer antigens and the intensive efforts that have been invested led to the successful development of a cancer vaccine for prostate cancer (32).

#### 2.1.4 T cell mechanisms of cancer cell detection

T cells play a crucial role in tumor immunosurveillance by recognizing and mounting adaptive responses to specific MHC-associated antigens displayed by malignant cells (33). T cells are divided into two main subsets: CD4+ helper T cells and CD8+ cytotoxic T cells (together known as conventional T cells). CD8+ cytotoxic T cells target directly and lyse cancer cells by secreting cytotoxic molecules such as perforin and granzymes (34); while CD4+ helper T cells catalyze an inflammatory environment, stimulate CD8+ T cells, NK cells, B cells, macrophages and DCs, promoting a predominantly Th1 anticancer response (35).

In contrast to the cytotoxic CD8+ and the helper CD4+ T cells, Tregs are believed to play an undesired role to which cancer progression has been linked (36). Tregs infiltrate tumors and express several anti-inflammatory cytokines (TGF-β, IL-10 and IL-35) that suppress the immune function of conventional T cells. CTLA-4 expression promotes the suppressive function of Tregs; and its blockade is shown to downregulate Tregs immune suppressive function (37). Another axis that is highly involved in promoting the suppressive function of Tregs is adenosine synthesis. Adenosine exerts highly suppressive functions on conventional T cells, incapacitating the desired inflammatory TME (38).

### 2.2 Immune response against helminths

#### 2.2.1 Innate immunity against helminths

Just as with the TME, the milieu where helminths reside is highly dependent on establishing an M2-enriched microenvironment allowing their survival. Helminth infection injures intestine epithelial cells and chemosensory cells (Tuft cells) in the gut (2, 39). Damage results in the release of DAMPs (2, 39), with tuft cells producing alarmins like IL-33, IL-25 and TSLP (40). These cytokines induce resident type 2 innate lymphoid cells (ILC2) to produce IL-4, IL-5 and IL-13 that together initiate a type 2 immune response (2, 39, 40). This response includes recruitment and activation of eosinophils, basophils, mast cells and M2 macrophages. M2 macrophages characteristically express Arg1, as well as RELM-α and the chitinase-like molecule Ym1. Important functions of M2 macrophages include the prevention intestinal microbiota translocation by promoting tissue repair (40). Moreover, they help to reinforce the intestinal barrier through mucus and anti-microbial peptide production and increase the clearance of dead enterocytes (39–41).

Neutrophils and eosinophils are important in early phases of infection. They recruit other effector cells and directly kill helminths especially at the larval stage (40). Nevertheless, helminths are resistant to innate immunity due to their large size and thick teguments. Additionally some of them have developed defense mechanisms against innate immunity (42).

#### 2.2.2 Adaptive immunity against helminths

Helminths induce predominantly Th2 protective responses (43). However, some intestinal helminths induce a initial Th1 response that rapidly shift to a Th2 phenotype (44–46). Intestinal DCs promote the differentiation of naïve CD4+ T cells into Th2 cells that in turn amplify the type 2 response through the secretion of IL-4, IL-13 and IL-5. While IL-4 stimulates the production of IgE leading to the activation of mast cells, IL-5 activates eosinophils (43). The type 2 cytokines, IL-4 and IL-13, promote clearance of the parasite through increased cell turnover, mucus production and increased peristalsis in the intestine (40, 41). They also induce the release of mast cells’ proteases that increase fluid secretion to the gut lumen, helping to “sweep out” the helminths. In addition, the type 2 cytokines promote M2 macrophage polarization (40).

In humans, peripheral blood analysis shows that helminth infection induces activation of both Th1 and Th2 responses, as well as activation of regulatory cells (40). The extent of the activation of each type of immune response defines the infection course, so in many cases a stronger Th2 response favors chronic infection, resistance to reinfection and lower burden of disease (40). Although this is not a general rule, since certain parasites like *Onchocerca volvulus* depend on the Th2 response to cause tissue damage (42).

However, helminths can resist the adaptive immune response through several mechanisms including, but not limited to, changes in surface antigens, development of a more resistant tegument, shedding of antigens or creating cysts and inducing a highly regulated Th2 response, known as a “modified Th2 response” (43) (**Figure 1B**).

#### 2.2.3 Immune regulation by helminths

Helminths can modulate the host immune response through anti-inflammatory changes in different immune cells as the infection becomes chronic. In most but not all helminth infections, these events are beneficial for both the parasite and the host, since the helminth ensures its survival and the host minimizes tissue damage, acquiring resistance to reinfection and avoiding allergic responses (42). One of the most studied mechanisms of immunomodulation by helminths is their capacity to modify DCs in a way that favors the generation of Tregs (39). This induction is probably driven by the helminth excretory/secretory (E/S) products and host cytokines, like IL-10 and TGF-β. In infected but asymptomatic humans, T cells show a cytokine profile that favors IL-4, IL-10 and TGF-β secretion over IL-17 and IFN-γ. When patients develop a symptomatic disease, the immune response is dominated by Th1 and Th17 responses (2, 40). The modified Th2 response induces the generation of non-stimulatory tolerogenic DCs (39) and M2 macrophages which together contribute to infection tolerance through their immunosuppressive and anti-inflammatory activities, i.e., inhibition of T cell proliferation and promotion of Treg differentiation (2). Treg expansion during helminth infection has been extensively studied in different helminth infections. This expansion decreases after antiparasitic treatment. Indeed, the depletion of Tregs in mouse models results in clearance of helminth infection. Tregs, therefore, contribute to the “modified Th2 response” (2).

Thus, helminth exposure seems to have a modulatory effect on the immune system. The interplay between the host response and helminth modulation allows the generation of a controlled inflammatory environment resulting in minimally symptomatic or asymptomatic chronic infections. Like in helminth infections, during cancer development, an initial pro-inflammatory anti-tumoral response predominates. As the tumor progresses, cells in the TME change to a regulatory phenotype. However, the regulatory environment that predominates in the TME results in further cancer growth.

## 3 Energetic requirements of immune cells

Immune cells depend on specific metabolic pathways to obtain energy, synthesize metabolites necessary for their growth and development, and initiate cell- or tissue-specific genetic programs. The cellular components of the immune system serve as a useful model for the study of metabolic adaptations due to their rapidly changing nature—as is the case with activated T lymphocytes, neutrophils, and macrophages—or, on the contrary, for their longevity and prolonged quiescence capability—when referring to naïve T lymphocytes and tissue-resident memory T cells (TRM). The “type” of metabolism adopted by a specific cell can also modify its longevity and effector capabilities (47).

In general terms, non-activated immune cells possess a catabolic phenotype, whereby pathways geared toward the production of energy in the form of ATP are mainly activated, such as fatty acid oxidation (FAO), which yields energy indirectly in the form of acetyl-CoA (48). Many of these quiescent cells also engage in so-called “futile” metabolic cycles, by which opposing pathways become active at the same time. A clear example of this are TRM. These cells have been primed before and now reside in tissues awaiting to reencounter the antigen to which they are specific. TRM engage simultaneously in FAO and β-reduction, processes which degrade and build fatty acids (FA), respectively. It is thought that this mechanism allows the TRM to maintain continuous activity of the enzymatic machinery needed to mount a quick and efficient immune response in the case of antigen presentation, and to “secure” a source of energy (48).

Naïve T cells are also quiescent and exhibit a low metabolic phenotype. Naïve T cells, contrary to TRM, do not (regularly) engage in futile metabolic cycles, depending almost entirely on FAO for energy acquisition. Likewise, these cells have a limited capacity to biosynthesize compounds required for their maintenance, and most of the FA that undergo FAO are obtained from the environment, as well as other biomolecules, such as glucose and glutamine. As such, because acetyl-CoA is a vital source of energy for the cell, the electron transport chain (ETC) and oxidative phosphorylation (OXPHOS), processes that produce ATP through a motor-proton gradient fueled by the sequential oxidation-reduction of various mitochondrial complexes, are highly active.

Upon encountering specific antigen, the T cell receptor (TCR) on the naïve T cell binds to the antigen-MHC dimer expressed by an antigen-presenting cell (APC). The formation of the immune synapse initiates a series of signaling cascades that activate the phosphatidyl-inositol 3 kinase (PI_3_K)-protein kinase B (PKB/AKT)-mammalian target of rapamycin (mTOR) axis (49, 50). The activation of these proteins, particularly mTOR, initiates a metabolic transition from catabolism to anabolism, compatible with the functions of an effector (activated) T cell (Teff). It is known that the activation of the adenosine monophosphate kinase (AMPK) pathway antagonizes this activation and favors an oxidative metabolism (51, 52). A crucial event in this transition is the augmented expression of glucose transporter 1 (GLUT1) on the cell membrane of activated T cells (53), a process which is also stimulated by IL-7 (54). The hallmark of anabolic activity in Teff is aerobic glycolysis, a seemingly paradoxical term that explains the method by which the rapidly proliferating Teff can satisfy its energetic necessities (53, 55–57). Glycolysis is, by definition, an anaerobic process that yields 4 molecules of ATP, 2 molecules of pyruvate and 2 molecules of NADH. In a hypoxic environment, pyruvate is shifted towards reduction to lactate by the enzyme lactate dehydrogenase (LDH). Lactate can be salvaged and turned back to glucose through gluconeogenesis, albeit this pathway is energetically expensive and rarely engaged by Teff. Despite its low ATP yield, aerobic glycolysis occurs rapidly, and allows for fast, if inefficient, obtention of energy. Thus, in Teff, mitochondrial oxidative activity becomes greatly reduced. Functionally, this translates to the fragmentation and reduction of mitochondria, as well as to a lesser rate of ETC and OXPHOS (49). Furthermore, aerobic glycolysis provides proliferating and activated cells with more advantages: it permits for the synthesis of metabolites that are crucial for the appropriate functioning of the cells (such as tricarboxylic acid (TCA) cycle intermediaries in the case of macrophages), a process known as anaplerosis (52); and allows for the indirect activation of pathways concerning transcription factor activation and cytokine synthesis.

Tregs are responsible for down-regulating immune responses to prevent immunopathology. Tregs share metabolic features with naïve T cells and TRM, in the sense that they mainly depend on FAO and oxidative metabolism (ETC, OXPHOS), rather than aerobic glycolysis (58, 59). Owing to this characteristic, Tregs scarcely express GLUT1 (60). FOXP3 is a transcription factor that is considered the master regulator of Treg development and function (61, 62). Thus, FOXP3 participates in the modeling of the metabolic landscape of Tregs once these cells become mature. FOXP3 increases oxygen consumption through OXPHOS induction; binds to and inhibits c-Myc mRNA (63), which is translated to a homonymous protein that upregulates genes related to glycolysis and glutaminolysis; diverts pyruvate to the mitochondria to avoid its reduction to lactate (58); and increases reducing agents (e.g., NADH) and components of the ETC (62). FOXP3 expression is necessary for the adoption of these metabolic adaptations; however, it seems that these metabolic features enforce FOXP3 expression as well, given that cultivating T CD4+ cells with the complex I inhibitor rotenone, greatly reduced FOXP3 expression and Treg proliferation (64). Alas, even if a genetic program is necessary for the acquisition of a particular metabolic phenotype, the “perpetuation” of said phenotype is also a prerequisite for its maintenance. A similar conclusion can be drawn from experiments conducted on Teff, which, when exposed to the glycolytic inhibitor 2-deoxyglucose (2-DG), reduced their effector function and expansion (64). Thus, signals derived from the presence or absence of substrates and the activation state of specific enzymes also determine the functionality and phenotype of an immune cell, a phenomenon termed “bottom-up” signaling (65). Bottom-up signaling is the rationale for various ongoing therapies that are attempting to override metabolic aberrations of immune cells in order to hone their effector function (in the case of cancer) or ablate it (in autoimmune disorders).

Macrophages are fascinating cells in both functional and metabolic terms, given that they can become polarized toward practically antagonistic states (21). M1 macrophages are pro-inflammatory (66) innate immune cells that secrete copious amounts of cytokines to activate and attract other immune cells. M1 macrophages also express inducible nitric oxide synthase (iNOS), an enzyme which converts arginine—an important modulator of macrophage polarization—to nitric oxide (NO) (51). NO can react with superoxide species to form peroxynitrite, an extremely potent microbicidal agent, used to eradicate pathogens phagocytosed by the macrophage. On the other hand, M2 macrophages, as previously mentioned, are anti-inflammatory, secreting IL-10 and TGF-β, and participate in tissue repair through the activation of fibroblasts. Contrary to M1 macrophages, the M2 subtype expresses Arg1 (67), an enzyme which degrades arginine into ornithine, which can be converted into polyamines, molecules with immunosuppressive properties (52, 67). M1 and M2 macrophages have different metabolic characteristics. M1 macrophages adopt an anabolic and glycolytic metabolic phenotype upon polarization, while M2 macrophages mainly employ oxidative processes in order to obtain energy. M2 macrophages take up triacylglycerols (TAG) from their environment through the scavenger receptor CD36 (67). The activation of proliferating peroxisome activating receptor (PPAR)-γ coactivator 1β (PGC-1β) is necessary for a successful polarization towards the M2 phenotype (67).

Lastly, DCs are responsible for activating naïve T cells and modulating their polarization. Quiescent (inactive) DCs that have not yet been stimulated by microbial antigens depend, like naïve T cells, on oxidative metabolism (mainly FAO) for their sustenance (56). When primed by ligand binding to either pattern recognition receptors (PRRs) or TLRs, DCs begin to perform aerobic glycolysis and activity of OXPHOS decreases (68), a process that is thought to be mediated, at least partly, by activation of the PI_3_K-AKT-mTOR pathway and the inhibition of the ETC by NO produced by iNOS (56).

## 4 Metabolic features of the tumor microenvironment

**Figure 2:**
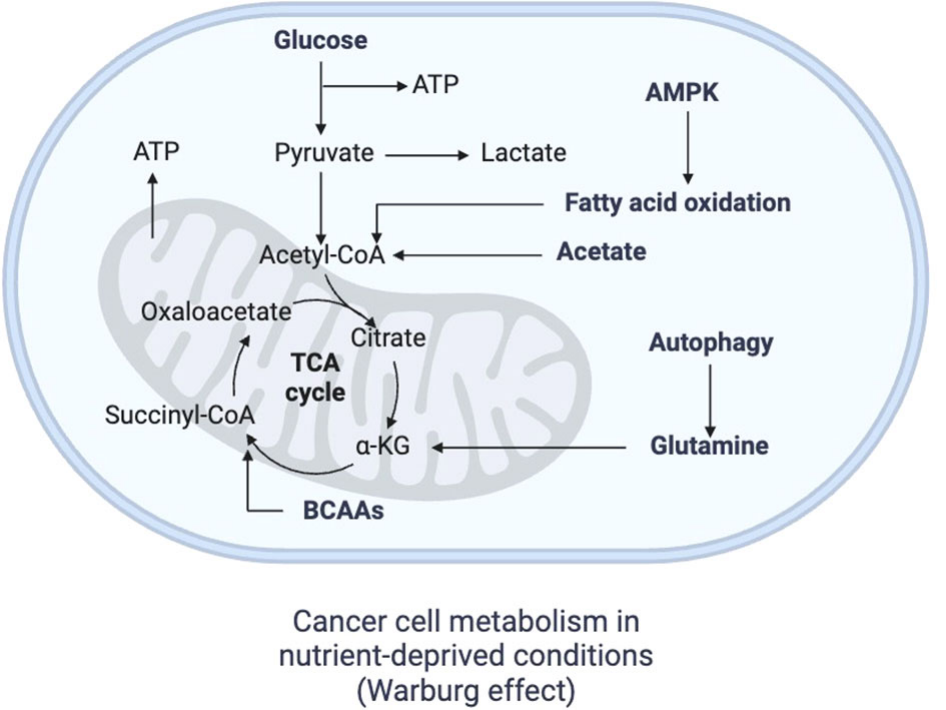
Metabolism of cancer cells. In the context of nutrient deprivation, cancer metabolism becomes highly glycolytic, producing lactate from pyruvate and avoiding mitochondrial metabolism, a phenomenon known as Warburg effect, which gradually occurs as the tumor grows and a nutrient-irrigation mismatch arises. FAO, fatty acid oxidation; TCA cycle, tricarboxylic cycle; AMPK, adenosine monophosphate kinase Created with BioRender.

Every microenvironment where the immune response is being executed has its own characteristics, but all of them tend to be acidic. An alkaline pH has not been reported for microenvironments where immune reactions happen (69). The TME represents the physical site where the neoplasia resides, and it consists of malignant cells, the surrounding extracellular matrix components, and additional cell populations such as fibroblasts, adipocytes, endothelial cells and infiltrated immune cells (70). Among immune cells within the TME, tumor-infiltrating lymphocytes (TILs), NK cells, myeloid-derived suppressor cells (MDSCs), DCs, and TAMs have been well studied (71). The TME has been classically seen as acidic, in part due to the high rate of replication by cancer cells with not enough blood perfusion, which makes some regions of the TME hypoxic and therefore unable to perform the regular OXPHOS for energetic purposes (72). The TME is especially acidic when malignant cells grow around basement membranes, which facilitates invasion and creates necrotic foci (69). Thus, cancer cells are forced to shift into a more glycolytic phenotype, producing lactate from pyruvate and avoiding mitochondrial metabolism. This route is not as energetically favorable, because while aerobic glycolysis only produces 2 molecules of ATP per molecule of glucose, OXPHOS provides 36 molecules of ATP instead (73). However, some cancer cells preferentially choose this pathway regardless of the oxygen concentrations in the TME, an event known as the Warburg effect (74), which provides tumors with energy in a fast way and with intermediates such as ribose-5-phosphate and glycerol for biosynthesis of nucleotides and lipids, respectively (73). Indeed, malignant cells are avid glucose consumers obtaining it from the TME through GLUTs, such as GLUT1 which is overexpressed in hypoxic environments by hypoxia-inducible factors (73) (**Figure 2**). Specifically, hypoxia-inducible factor 1α (HIF-1α) mediates the transcriptional program facilitating cancer cells to switch from OXPHOS to glycolysis (75). For glucose to be transformed to lactate, enough supply of LDH is provided in cancer cells (76). Since lactate can induce damage to cancer cells, its accumulation is prevented through the overexpression of the proton-linked monocarboxylate transporters (MCTs), especially MCT1 and MCT4, secreting it into the TME and simultaneously dropping the pH (73). In fact, the pH shifts from around 7.4 in serum to 6.2-6.8 in the TME, which implies an increase in H+ concentration from ~40nM to ~630-160nM, respectively (77). By avoiding lower intracellular pH levels, cancer cells prevent a reduction in the rate of cellular processes that are also required for survival, such as DNA and protein synthesis, and even mitosis (69).

### 4.1 The immune response against cancer cells is hampered by the acidic pH and by energetic limitations in the microenvironment

**Figure 3:**
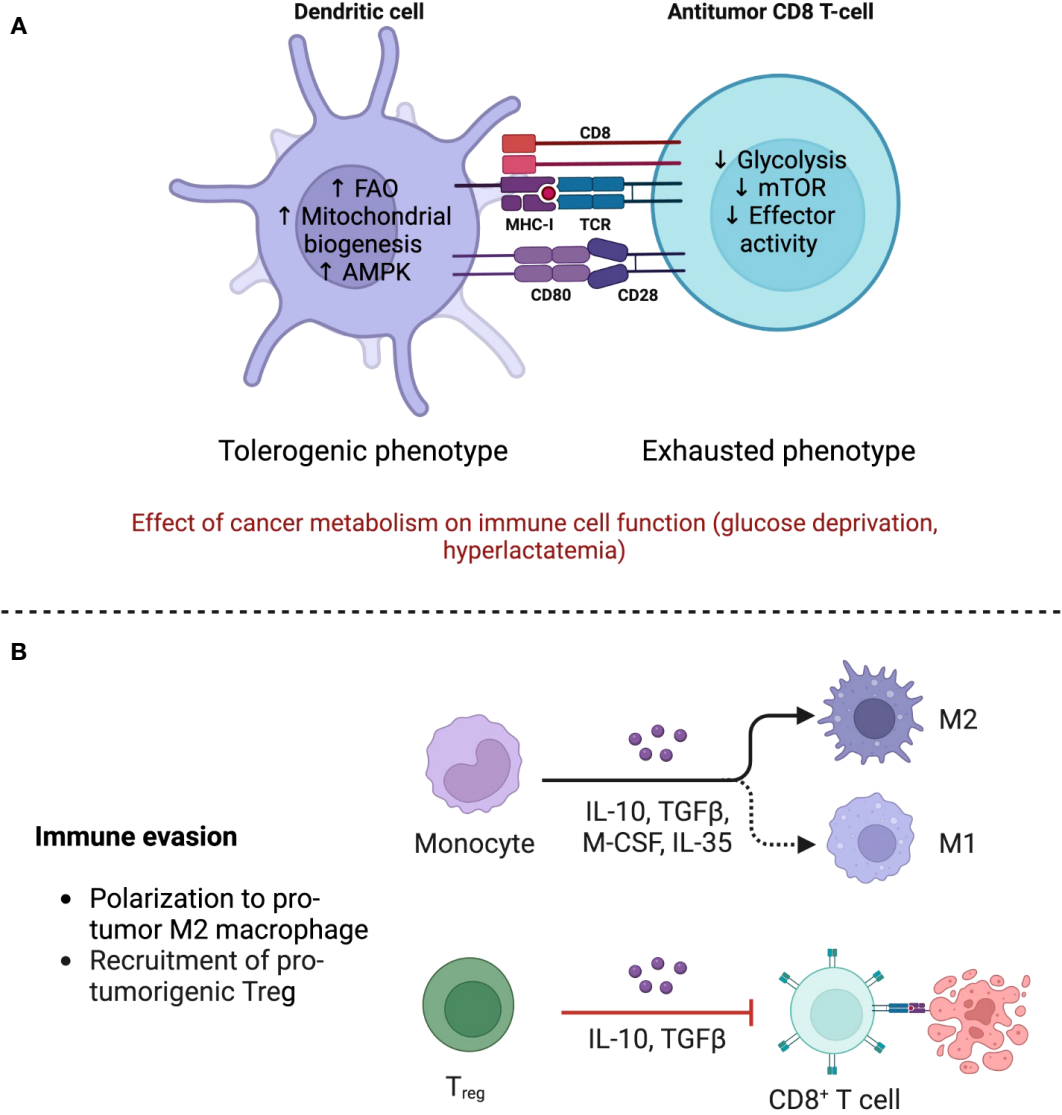
The TME shapes the phenotype of immune cells through immune and metabolic processes. **(A)** The anti-tumor response is mainly mediated by Tregs, which maintain an oxidative metabolism even in the nutrient deprived TME, allowing them to strengthen their suppressive functions. Polarization of monocytes toward M2 macrophages also contributes to the dampening of the immune response by secreting immunomodulating cytokines such as IL-10 and TGF-β. Adapted from “The Tumor Microenvironment: Overview of Cancer-Associated Changes” by BioRender.com (2023), https://app.biorender.com/profile/FarahRamadan96/templates/5f9834cd023b8300a2284c9b. **(B)** Metabolism of immune cells in the TME. Dendritic cells (DCs) acquire a tolerogenic phenotype associated with diminished priming, while T CD8+ cells, in addition to being less stimulated by DCs, become exhausted due to glucose scarcity and mTOR downregulation. FAO, fatty acid oxidation; AMPK, adenosine monophosphate kinase; mTOR, mammalian target of rapamycin. Created with BioRender.

Several immune cells, including neutrophils, macrophages and T lymphocytes, are highly plastic and polarize to pro- or anti-inflammatory phenotypes depending on the environment. This is also true for other infiltrated immune cells within the TME. The influence of immune mediators such as TGF-β within the TME promotes the generation of neutrophils with a N2 phenotype, which are pro-tumoral, and the existence of type I IFN such as IFN-β reduces the presence of N1 neutrophils that are anti-tumoral (78). Regarding TAMs, the “classically activated” M1 anti-tumoral macrophages, whose differentiation is based on stimuli by lipopolysaccharide (LPS), TNF-α and IFN-γ, are replaced by the “alternatively activated” M2 pro-tumoral macrophages, which are pro-angiogenic and depend on IL-4, IL-10, PGE2 and TGF-β (79). In the case of TILs, even though they are the main effectors of the anti-tumoral immune responses, it is possible that, as the malignancies progresses, the presence of IFN-γ-producing Th1 cells reduces while the immunosuppressive Tregs, which rely on IL-2, IL-10, TGF-β and IL-35, increase (80). Taken together, the N2 neutrophils, M2 macrophages and Tregs enable the persistence and dissemination of the malignancy (**Figure 3A**).

DCs also become dysfunctional. Purportedly, immunosuppressing factors in the TME, such as IL-10, TGF-β, and Wnt5, favor the adoption of a tolerogenic phenotype through a β-catenin-mediated mechanism, whereby IDO expression is increased (56). Furthermore, both Treg and tumor cells express programmed death-ligand 1 (PD-L1) and CTLA-4. The first binds to programmed cell death protein 1 (PD1) on the surface of Teff, initiating an exhaustion program that compromises aerobic glycolysis (48). CTLA-4 blocks co-stimulation by CD80/CD86 by acting as a decoy of CD28 (81). This effectively abrogates the “second signal” in T cell activation and induces anergy (**Figure 3B**).

As Lardner A. reviewed before, the acidic extracellular pH strongly affects the activity and behavior of immune cells. Normally a drop in pH up to 6.5 is not able to impede locomotion of leukocytes in a significant way, suggesting that infiltrated immune cells still should be able to migrate to the TME and to mobilize within it. However, under acidic pH, neutrophils see their oxygen consumption reduced up to 90%, their tumor cytotoxicity and hydrogen peroxide (H_2_O_2_) production is limited, and their phagocytic activity is suppressed (69). Macrophages, on the other hand, release reduced amounts of TNF-α (69), IL-6, IL-10 and NO when there is a low extracellular pH (82). Also in macrophages, even though the secretion of both IL-6 and IL-10 diminishes upon low pH environments, IL-10 reduction is even higher, yielding a ratio of IL-6:IL-10 from 5:1 at pH 7.4, to 55:1 at pH 6.5 (83). In the case of lymphocytes in acidic microenvironments, IL-2 fails to work as a proliferation inductor, and such cells have a limited lytic activity against cancer cells (69).

Besides, an acidic TME directly stimulates immune evasion by malignant cells. For example, the increased presence of lactate in the TME significantly enhances the suppressive activities of MDSCs through the HIF-1α pathway (75). When activated, MDSCs rely on aerobic glycolysis for ATP synthesis. However, in the TME, a shift to oxidative metabolism is favored, and the MDSCs up-regulate FAO and OXPHOS greatly. This transition enforces their suppressive functions, many of which pertain to nutrient deprivation, such as arginine and cysteine (51). Along with Treg consolidation in the TME, these processes contribute to the inefficiency of the anti-tumor response (75). On the other hand, Kwon YJ et al. reported in the triple-negative breast cancer cell line MDA-MB-231 that extracellular acidosis promoted the expression of PDL-1 up to 4 times, an effect that was reverted upon returning to a normal range of pH or with the LDH inhibitor oxamate (76). Upregulated PDL-1 levels in non-small cell lung cancer (NSCLC) tumors have been associated, not only with the cancer stage in patients, but also with glucose uptake by such cells (84). A high PDL-1 expression by cancer cells is commonly seen in advanced tumors, and it binds to PD-1 in infiltrated immune cells, mainly to suppress the mTOR pathway in macrophages and to induce exhaustion in cytotoxic T lymphocytes, which eludes the anti-tumor action by immune cells (70). Additionally, an acidic pH of 6.5 disrupts IL-2 signaling by reducing STAT5 phosphorylation in pre-activated CD8+ T cells (85). STAT5 is a critical mediator for CD8+ T cell responses, since it acts as a signal transducer for the CD8+ T cell pro-survival cytokines, IL-7 and IL-15 (86). However, for Tregs even low doses of IL-2 could secure their survival and maintenance (87). Thus, it is possible that the acidic pH in the TME does not only stimulate the exhaustion of TILs, but also that by Treg enrichment and survival, it limits the presence of ATP that could be used by the newly arrived immune cells to ensure their proper anti-tumor activities. The implications of this statement deserve further investigation.

More recently, it has been shown that changes in pH within the TME could be working together with the limitations in energetic resources, to induce phenotype shifts on immune cells. Such a hostile environment depleted of nutrients, particularly glucose, demands energetic plasticity from immune cells. Certainly, while a reduced availability of glucose strongly limits glycolysis and the capacity of T cells to proliferate and to produce cytokines, minor concentrations of glutamine diminish T cell activation (49) (**Table 1**). The restriction in nutrients within the TME has led to the concept of “metabolic competition”, and now it is known that the predatory consumption of glucose by malignant cells metabolically restricts TILs. Indeed, by using a mice model of sarcoma, the group led by Pearce E. has demonstrated that CD8+ T cells in contact with such cancer cells had a limited aerobic glycolysis capacity and produced low levels of IFN-γ, which correlated with the glucose concentration in the media. Importantly, while the relative presence of Tregs was high, the M1:M2 ratio was low in the TME of the tumors (94).

**Table 1:** The modulation of the metabolism in immune cells improves their anti-tumor activity.

Targeted metabolites	Cancer models	Treatments used	Immune effects	Biological effects	References
Glutamine	Subcutaneous inoculation of the cell lines MC38 (colon cancer), CT26 (colon cancer), EL-4 (lymphoma), or B16 (melanoma) in mice	JHU083 (a prodrug of the glutamine antagonist DON)	*In vivo*: Efficacy dependent on CD8^+^ T cells; induction of protective memory, as tested by rechallenging after mice were cured; efficacy; enhanced function when used in combination with PD-1 immunotherapy; marked increase in infiltrated CD8^+^ T cells, which by RNAseq demonstrated to be highly proliferative, activated and less exhausted; energetic phenotype of T cell memory with upregulation in OXPHOS	*In vivo*: Decrease in tumor growth and promotion of mice survival; disabling of the Warburg effect and ablation of both glycolysis and OXPHOS on cancer cells; decrease in tumor hypoxia; increase in glutamine and glucose availability in tumors	(88)
Glucose	Subcutaneous inoculation of RCC cells with inactivation in the von Hippel-Lindau tumor suppressor in mice	STF-31 (GLUT1 antagonist)	No immunosuppression by the treatment *in vivo*. CD4^+^ T cells do not reduce their ECAR at concentrations up to 100μM	*In vitro*: Malignant viability reduction in a dose-depended manner via necrotic cell death; inhibition of hexokinase activity, lactate production and extracellular acidification. *In vivo*: decrease in glucose uptake in tumors by FDG-PET, with delay in tumor growth without hemolysis or other signs of systemic toxicity	(89, 90)
Butyrate	Subcutaneous inoculation of MC38 (colon cancer), CT26 (colon cancer), or MCA101_OVA_ (fibrosarcoma) in mice	Anti-CTLA-4 ± supplementation with butyrate	*In vivo*: In DCs, butyrate impeded the stimulation of CD80 and CD86, as well as the expression of MHC-II, that was promoted by the anti-CTLA-4 treatment; the upregulation in IFN-γ in CD8^+^ T cells treated with anti-CTLA-4 was inhibited when butyrate was added	*In vivo*: butyrate abolished the anti-tumor effects of the anti-CTLA-4 treatment	(91)	
Glutamine	Orthotopic inoculation of the TNBC cell line E0771 in mice	V-9302 (glutamine transporter inhibitor)	*In vivo*: No significant impact on total numbers of CD45^+^ or CD45^+^CD3^+^ T leukocytes; deeper tumor infiltration of CD8^+^ T cells, which also were positive for activation markers (granzyme B, CD107a, IFN-γ, CD25, CD69, and CD44); downregulation in Treg populations in tumors. *Ex vivo*: Antigen-directed cytotoxicity against malignant cells and stimulation in the uptake of glutamine by CD8^+^ T cells; increase in the effector memory T cell population (CD44^+^CD62L^+^)	*In vivo*: reduction of tumor growth and weight, with induction of apoptosis	(92)	
Cholesterol	Intravenous injection of B16 (melanoma), or subcutaneous inoculation of LL2 (Lewis lung carcinoma) or MC38 (colon cancer) in mice	β-cyclodextrin or simvastatin (cholesterol-depleting agent), or shRNA against *Hmgcr*	*Ex vivo*: When cholesterol levels in CD8^+^ T cells were low, there was an improvement in inhibitory receptors related to T cell exhaustion (PD-1, TIM-3, LAG-3, 2B4); apoptosis was reduced; migration was improved; OXPHOS and glycolysis increased; and the anti-tumor activity was restored as measured by granzyme B, IFN-γ and TNF-α production in CD8^+^ T cells. *In vivo*: CD8^+^ TIL from shRNA-treated cancer cells against *Hmgcr*, or with simvastatin, had lower PD-1 and 2B4 expression, and less cholesterol content, with better anti-tumor activity	*In vitro*/*in vivo*: Cholesterol-treated CD8^+^ T cells had upregulation in lipid-metabolism related genes, and especially in the ER-stress-response genes such as *XBP1*. *In vivo*: Cholesterol depletion reduced the tumor volume and the number of tumor foci	(93)	

Acronyms, DON, 6-diazo-5-oxo-l-norleucine; OXPHOS, oxidative phosphorylation; RCC, renal cell carcinoma; GLUT1, glucose transporter 1; FDG-PET, fluorodeoxyglucose positron emission tomography; ECAR, extracellular acidification rate; CTLA-4, cytotoxic T-lymphocyte antigen 4; DCs, dendritic cells; MHC-II, major histocompatibility complex II; TNBC, triple-negative breast cancer; ER, endoplasmic reticulum; shRNA, short hairpin RNA.

Just as with glucose, glutamine utilization by immune cells in the TME is restricted. Given the pleiotropic routes of glutamine as an anaplerotic source for OXPHOS and as an intermediate for the biosynthesis of other molecules, some tumor cells are highly dependent on it and termed “glutamine addicted”. The detailed description of the glutamine metabolism in cancer cells is outside of the scope of this review, but outstanding explanations of this topic can be seen in (95, 96). Briefly, it is known that activated T cells require both a high glycolytic rate and glutamine metabolism to ensure fast energy, to proliferate and synthesize proteins, and in fact, their ratio of glutamine intake is similar or even higher than that of glucose (97). Among other roles in lymphocytes, glutamine is required for the expression of surface markers, for proliferation, and for the production of IFN-γ, TNF-α and even IL-6 (97). Under glutamine deprivation states, such as in the TME, TCR-activated naïve CD4+ T cells differentiate to Tregs through the inhibition of the mTOR activity, and just as under low glucose concentrations, the generated Tregs demonstrate low levels of glycolysis concomitantly limiting their ATP levels (98). By reducing the presence of glucose and glutamine in the TME, effector TILs are unable to proliferate and to exert anti-tumor activities, and instead are differentiated into Tregs to maintain a tolerogenic pro-tumor environment. Thus, the induction of Tregs is also favored by the TME. Tregs do not rely on glucose for ATP production, and they are highly resistant to the toxic effects of elevated lactate concentrations. As such, Tregs are capable of surviving and even thriving within the TME.

It is not surprising that infiltrated immune cells must reprogram their metabolism according to the presence of FA, glutamine and glucose in the TME (**Table 1**). There is a particular interest in the research of lipids, because both cancer cells and cancer-associated fibroblasts (CAFs) within the TME activate adipocytes to lipolyze their triglyceride depots (99), making the TME particularly enriched in lipids. Importantly, some malignant cells such as those from breast cancer have been suggested to exist in a somewhat “parasitic” association with adipocytes and the lipids inside them, and when adipocytes are activated, they also secrete IL-6 which then activates STAT3 and ultimately the membrane lipid transporter CD36 (99). IL-6 facilitates the recruitment of T cells into the TME but also drives chronic inflammation in cancer, and its overexpression is seen as a prognostic indicator of poor outcomes for cancer patients because it drives tumor progression through the activation of the cell cycle regulator cyclin D1, the proto-oncogene c-Myc, and the master metabolic regulator mTOR complex 1 (mTORC1) (100). TILs, such as those found within the TME, have been shown to upregulate their CD36 and their lipid chaperone FABP4/5, both of which ensure the uptake of exogenous lipids (101). Lipids being used by T cells represent a hallmark of Tregs, which then use them as a fuel for OXPHOS through FAO (102). In fact, the transcription factor PPAR-γ, which promotes the transcription of FOXP3 and therefore induces the establishment of Tregs, also stimulates FAO in Tregs (102). This series of events are maintained by IL-2, which just like IL-6, activates mTORC1 signaling in Tregs (103). mTORC1, in turn, couples an increase in the metabolism of cholesterol and other lipids and ensures the expression of the immunosuppressive molecule CTLA-4 on Tregs (48, 103). Thus, the TME shapes the metabolic adaptations of immune cells and impacts tumor development.

## 5 Metabolic features of the parasite infection site

Regarding parasitic infections, less concise information is available about the microenvironment where they reside. Studies on the metabolism of immune cells in sites of helminth infection are just beginning to emerge. This is in part due to their highly complex life cycles, which are characteristic of each parasite. Furthermore, depending on their life stage, they tend to escape to different tissues and organs within the infected organism. Helminths are multicellular organisms and do not reside intracellularly. Since helminths are more likely to inhabit in considerably bigger spaces, such as mesenteric veins and the middle third of the jejunum for the adult forms of *Schistosoma* and *Ascaris*, respectively, the idea of a “microenvironment” for such parasites would be too short. Instead, we could define the sites of helminth infection as infected regions. In general, the migration of helminths through tissues stimulates both Th2 and regulatory immune responses, which is an advantage mechanism used by the larval stages of parasites that colonize such areas (104). Helminths not only affect the surrounding immune cells but can also affect the systemic immune response.

### 5.1 The plasticity of immune cells is altered in helminthic infections due to energetic requirements

**Figure 4:**
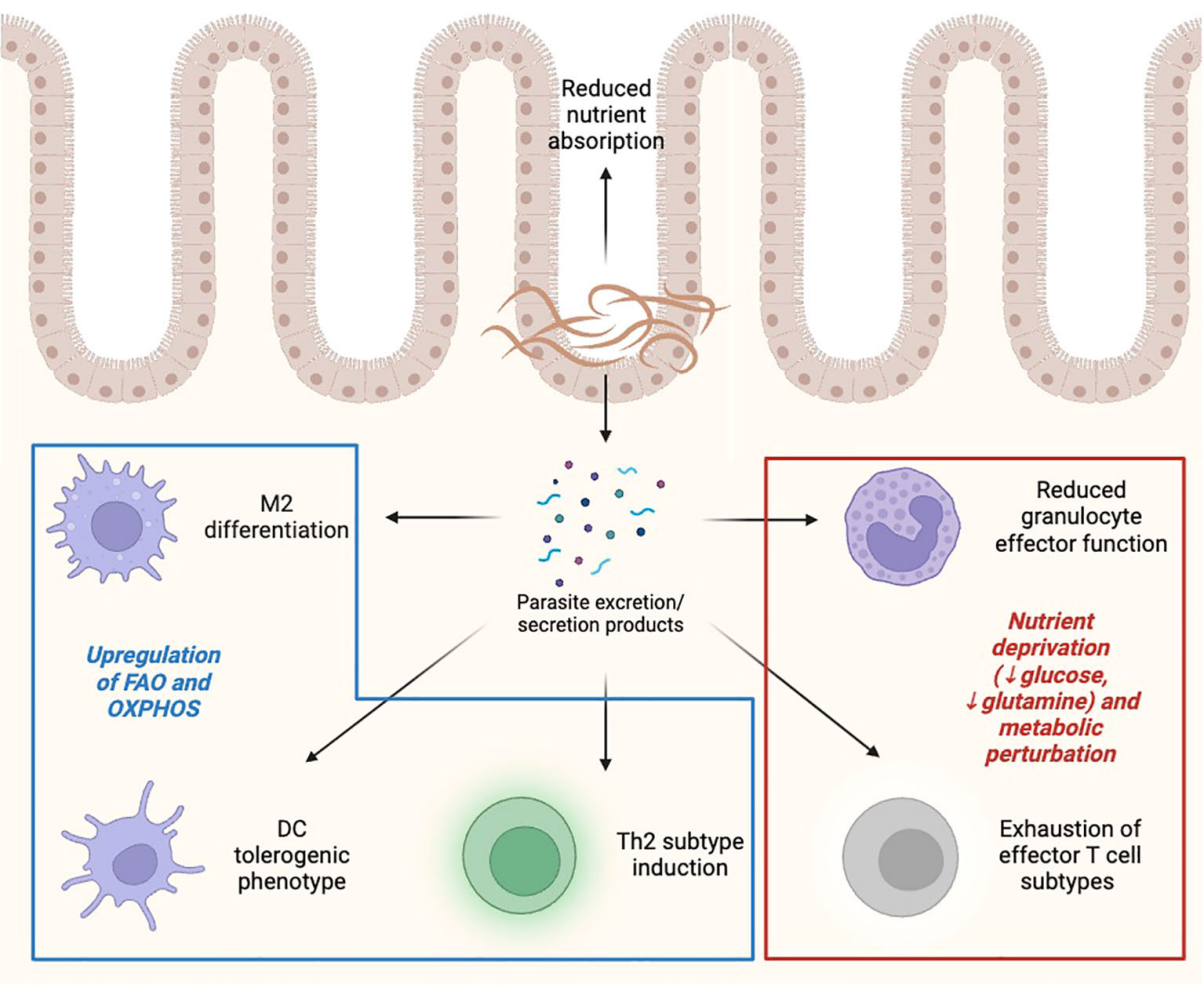
Effect of helminths on immunometabolic landscape. Helminths, either directly or through the production of excretion/secretion products, induce metabolic and immune alterations in effector cells, including promoting M2/Th2 polarization, reducing effector capacity of inflammatory innate and adaptive cells, and limiting nutrient availability to rapidly proliferating cells. FAO, fatty acid oxidation; OXPHOS, oxidative phosphorylation. Created with BioRender.

Changes in metabolism are crucial in granting immune cells plasticity, that is, the ability to change functional activity depending on a given situation. Helminths, like tumor cells, can modify metabolic features of infiltrated immune cells. For example, M1 macrophages under the activation of PRRs, like the TLR4 by LPS, increase the expression of HIF1-α and therefore obtain energy in a fast way through glycolysis (5). However, IL-4, a marker of type 2 immune responses, promotes OXPHOS and mitochondrial respiration in M2 macrophages surrounding the parasite. Helminths promote IL-4 production and M2 differentiation (5, 105) (**Table 2**). Less glycolysis implies a reduced availability of intermediates for nucleotide synthesis, and thus less capacity for replication in macrophages (**Figure 4**). In some sense, certain similarities can be seen when comparing the infected regions by helminths against the TME. In both cases, the M1/Th1 phenotypes are blocked, in detriment of the energetic requirements of infiltrated immune cells.

**Table 2:** Metabolic effects on immune cells impact immune regulation.

Parasite infection or stimuli	Treatment or model	Metabolic effects in immune cells	Metabolic effects at tissue and systemic level	References
Secretion of IL-4	—	Promotion of OXPHOS and mitochondrial respiration in M2 macrophages surrounding the parasite	—	(5)
Activation of M2 macrophages with IL-4	Bone marrow-derived macrophages isolated from femurs and tibias, stimulated with murine IL-4	Enhanced FAO promoted by STAT6 and PGC1β. Inhibition of proinflammatory cytokine production	—	(5, 106)
IL-4R signaling in M2 macrophages	Myeloid-cell-specific IL-4Rα-deficient mice (*Il4ra* ^fl/−^ *Lyz2-cre*) and *Retnla* ^−/−^ mice	M2 macrophages activated by IL-4R signaling express IGF-1, RELMα and Arg-1. RELMα and Arg-1 enhance wound healing.	—	(104, 107)
Stimulation with IL-4	CD36 expression induced by IL-4 in RAW 264.7 cells	Induction of CD36 expression and lysosomal function with endocytosis of LDL and VLDL	—	(108)
*Brugia malayi*	Human monocyte-derived dendritic cells exposed to *B. malayi* microfilaria from infected jirds	Downregulation of components of the mTOR signaling pathway in microfilaria-induced DC. Inhibition of mTOR and its regulatory proteins phosphorylation which are vital for protein synthesis in DC. Increased autophagy	—	(109)
*Nippostrongylus brasiliensis*	RIP2-Opa1KO mice with pancreatic β cell Opa1 deficiency, infected trough subcutaneous inoculation of third stage *N. brasiliencis* larvae	—	Increase in WAT eosinophils and M2 macrophages with increased expression of M2 markers (YM1 and Arg-1). Increase in body insulin sensitivity and glucose tolerance, diminished hepatic steatosis	(41, 110)
*Heligmosomoides polygyrus*	C57BL/6 mice fed with high-fat diet and infected with 200 third stage *H. polygyrus* larvae	—	Decreased weight gain, increase in glucose tolerance and WAT beiging. Increase in WAT M2 gene expression and M2 markers	(41, 111)
*Fasciola hepatica*	Bone marrow-derived macrophages and peritoneal macrophages from C57BL/6 mice, stimulated with synthetic FhHDM-1	FhDHM-1 reprograms macrophages by inducing OXPHOS and elevation of glutaminolysis, resulting in inhibition of pro-inflammatory cytokines (TNF and IL-6) independent of M2 polarization. Inhibition of lysosomal vATPSase.	—	(112)
*Schistosoma mansoni*	Arg1(-/flox); LysMcre mice and Arg1(flox/flox);Tie2cre mice	Macrophage expression of Arg-1, triggered by *S. mansoni* infection, downregulates inflammation and T proliferation by depleting arginine concentrations. Arg-1 expressing macrophages have an anti-fibrotic activity during Th2 response, and are important mediators of immune modulation of chronic schistosomiasis	—	(113)
*Heligmosomoides polygyrus*	*A*_2B_ *AR* ^−/−^ BL/6 mice infected with third stage *H. polygyrus* larvae trough oral administration	Adenosine initiates a helminth-induced type 2 response through interaction with the A2B adenosine receptor. Upregulation of IL-33 and the subsequent activation of ILC2 cells	—	(104, 114)

Acronyms, OXPHOS, oxidative phosphorylation; Arg-1, arginase 1; WAT, white adipose tissue; RELMα, resistin-like molecule alpha. FhHDM-1, *Fasciola hepatica* helminth defense molecule; DC, dendritic cell. mTOR, mammalian target of rapamycin. —, Not determined.

Although M2-like regulatory macrophages develop in both the tumor and helminth environments, in the TME conditions of hypoxia and glycolysis usually prevail, while in helminth infection sites, IL-4 causes activation of OXPHOS, suggesting that regulatory TAMs and helminth-derived M2 macrophages may have different metabolic needs. Indeed, different helminth-derived molecules use diverse mechanisms for metabolic reprograming. A helminth product secreted by the liver fluke *Fasciola hepatica*, the helminth defense molecule-1 (FhHDM-1) can reprogram macrophages by inducing OXPHOS fueled by FA with a concomitant elevation of glutaminolysis that result in inhibition of pro-inflammatory cytokines independent of M2 polarization, as no changes in M2 markers were observed. Besides, FhHDM-1 inhibits lysosomal vacuolar ATPase in contrast to IL-4 metabolic reprogramming (107). In contrast, IL-4-induced M2 differentiation depends on FAO, specifically lysosomal lipolysis through activation of lysosomal acid lipase (LAL) and by intake of FA by the scavenger CD36 for activation of OXPHOS and M2 polarization. Furthermore, IL-4 is a potent inductor of CD36 expression and lysosomal function, which is responsible for endocytosis of low-density lipoproteins (LDLs) and very low-density lipoproteins (VLDLs) (108). The lipid lysophosphatidylcholine from *S. mansoni* can induce M2 polarization in a PPARγ‐dependent manner (115). This is relevant in the context of metabolic reprogramming since PPARγ is induced by IL-4 and is considered a link between M2 macrophage activation and glutamine metabolism (116). Infection with *S. japonicum* or its products, like soluble egg antigens up-regulate mRNA of enzymes involved in FA synthesis and oxidation, and thus promote M2 polarization (68, 117). In this case, inhibition of lipolysis results in diminished protective immunity in a mouse model of helminth infection (**Table 2**).

FA synthesis and mitochondrial function are important for DC generation and immune phenotype (118). Tolerogenic DCs are dependent on glycolysis, OXPHOS and increased FAO. Helminth infections are associated with tolerogenic DCs, and similarly to helminth-derived M2 macrophages, OXPHOS and FAO are upregulated (119). Microfilariae from the nematode *Brugia malayi* can inhibit phosphorylation of mTOR, which is involved in FA synthesis and oxidation, and interfere with DC function, suggesting that this mechanism could also promote the differentiation of Tregs (109) (**Table 2**).

Glutamine deprivation is significant in the context of parasitism. In helminths, glutamine acts as an important metabolite for production of energy, mainly through gluconeogenesis, as well as a regulator of the synthesis of nucleic acids (120). Motility of multicellular parasites was enhanced when grown in a glutamine-rich medium and reduced when the amino acid was depleted (121). A role in the defense against xenobiotics and other stressors has been suggested for glutamine, given that glutamine synthase, the enzyme which catalyzes the conversion of glutamate and ammonia to glutamine, is upregulated in *S. japonicum* specimens exposed to antiparasitic drugs (122). Helminths have also been shown to skew the immune response in their favor by modifying glutamine metabolism. In a *F. hepatica* model, the use of the peptide FhHDM-1 resulted in accentuated glutaminolysis in macrophages (107), resulting in a tolerogenic phenotype that favored parasite survival.

Activation of T cells, with concomitant proliferation and cytokine production, is energy-dependent and consumes glucose. Indeed, T cells over-express GLUT1. OXPHOS and cytochrome c in mitochondria, as well as glutamine import, are elevated upon activation (123). Under glutamine deprivation T cells differentiate into Tregs. This has been shown in the TME as well, where activated CD4+ T cells differentiate to Tregs through the inhibition of its mTOR activity (98). FAO, on the other hand, modulates effector T cells versus Treg generation, favoring the latter (124). These findings are consistent with the adipose tissue decrease observed in helminth-infected obesity mouse models (see below).

T cell exhaustion is not only characteristic of the immune cells found in the TME. Exhaustion has been reported in microbial infections as well, limiting the ability of immune cells to control pathogens (124). However, in helminth infections, T cell exhaustion has been poorly studied. One study showed that most *Wuchereria bancrofti*-infected individuals have asymptomatic disease. However, some patients develop lymphedema, characterized by constant immune activation and exhausted CD8+ T cell phenotypes (125).

ILC2 are important innate immune cells induced during helminth infections. Due to their size and nutritional requirements, helminths can compete for nutrients with the host. Helminths use glucose as their main nutritional source. As mentioned throughout this review, helminths impact, not only glucose metabolism but also FAO and amino acid metabolism, and affect ILC2 and other immune cell functions. ILC2 catabolize externally derived FA through FAO to induce OXPHOS and ATP synthesis. The use of internal FA, derived from autophagy, also fuels this pathway in mitochondria. In addition, mTOR controls amino acid metabolism that supports ILC2-mediated immune responses (126) (**Table 2**).

From an intuitive perspective, one way in which parasites could alter the metabolism of the immune cells is through nutrient depletion. Multicellular parasites, such as gastrointestinal nematodes, have been shown to divert nutrients and modify the host’s appetite in order to promote their development and growth (127). *Heligmosomoides polygyrus*, for instance, reduces glucose transport across the intestinal mucosa, ensuring the monosaccharide’s availability in the intestinal lumen (128).

Thus, an additional convergence point can be found between the restriction in nutrients within the TME that implies “metabolic competition” between cancer and immune cells as mentioned above, and the “nutritional competition” posed by the large parasite and the surrounding immune cells. In both cases, restrictions of immune cell physiology and differentiation cues need to adapt to the microenvironment.

### 5.2 Effect of helminth infections on host metabolism

In rodent models, helminth infection and their products can induce changes in global metabolism. Some of the modifications include improvement in insulin sensitivity and glucose tolerance, decrease in body weight, decrease in hepatic steatosis, white adipose tissue (WAT) beigeing, and an increase in WAT eosinophils and M2 macrophages with increased expression of M2 markers (Ym1 and Arg1). However, in humans there is no evidence of causality of the metabolic changes. The main associations observed through epidemiological cross-sectional studies in endemic areas, are the decrease in the homeostatic model assessment for insulin resistance (HOMA-IR), reduced prevalence of metabolic syndrome, obesity and type two diabetes mellitus (T2DM) (41). These results suggest that helminth infection could improve insulin sensitivity and glucose homeostasis, two aspects strongly related to cancer development.

There is still no information on the cellular mechanisms by which helminths induce these metabolic changes in humans. Diverse studies in rodents show that the metabolic changes induced by helminth infection and their secretory/excretory products, could be due to a change in microbiota species and eosinophil and M2 accumulation in mesenteric lymph nodes, adipose tissue, small bowel and liver (129). However, the possible mechanisms involved in these metabolic changes largely remain unknown, as well as the direct role of eosinophils and Th2 cytokines on insulin resistance.

Some parasitic infections in mouse models regulate adipogenesis. For example, *Echinoccoccus granulosus* infection promotes lipolysis and *H. polygyrus* attenuates obesity at least in part through enhanced arginine metabolism and PPAR-γ pathway activation that are associated with M2/Th2 polarization in adipose tissue contributing to an anti-inflammatory environment in infected mice (111, 124). Nevertheless, the effects of helminth infection on carcinogenesis have contradictory results, with some studies showing protection of cancer development, while others have either no effect or cancer-promoting activities. These discrepancies may be due to the different helminth species used in the studies, as well as the timing of infection and tumor progression (130).

Regarding the changes in immunometabolism, a broadly studied phenomenon is macrophage modifications by *Schistosoma* products (mainly eggs and their related antigens) that reprogram metabolism-related genes when they drive a M2 phenotype in rodent models. *Schistosoma* up‐regulates FAO and diminishes lipid accumulation in the liver through the up‐regulation of AKT and mTORC1 (modulators of catabolism and glucose metabolism). Additionally, this macrophage reprogramming seems to bring protection from high‐fat diet induced weight gain, type 2 diabetes and atherosclerosis (116). Further studies are needed to understand the mechanisms involved in helminth-induced metabolic changes that have potential therapeutic value.

## 6 Improving the immune activity by targeting the metabolic aberrations in cancer and in helminthic infections

Numerous recent therapeutic approaches have been focused on correcting the metabolic dysregulation seen in effector cells within the TME. PD-1 and CTLA-4 antagonists, whose agents belong to the category of immune checkpoint blockers, have been shown to partially correct these abnormalities (52). PD-1 signaling, for example, blocks activation of the PI_3_K-AKT-mTOR axis, while CTLA-4 inhibits glycolysis (47). Thus, by antagonizing these receptors, the quiescent metabolic phenotype caused by their binding to their ligands is abrogated (49). Atypic immune checkpoints could also serve as targets for future immunometabolic therapies. Clever-1, for instance, is a scavenger receptor that acts as a checkpoint of macrophages and other immune cells (131). Blocking Clever-1 with bexmarilimab, polarizes macrophages to an M1 phenotype cells. This strategy showed promise in activating T cell responses and improving anti-tumor activity (132).

Broadly, metabolic reprogramming could also “orient” an immune response in a particular direction. Immunosuppressive cancers would require glycolysis stimulation and FAO inhibition (133). Metformin, a complex I inhibitor used to treat insulin resistance and T2DM, may act on immune cells by reverting dependence on ETC and OXPHOS and stimulating a shift toward glycolysis. Indeed, metformin treatment prevented apoptosis and exhaustion of CD8+ TILs (54). Addition of substrates that reverse the exhausted phenotype of Teff in cancer, such as glutamine and pyruvate (134), could also prove beneficial (135). Generation of TILs that depend on FAO for energy production, but do not lose effector anti-tumor function, can be accomplished with PPAR-α agonists (134), opening the possibility for induction of metabolically “atypical” Teff that are well acclimated to the TME conditions. Increasing cholesterol availability through inhibition of acetyl-CoA acetyltransferase 1 stimulated TCR clustering and signaling (136), in a sense increasing sensitivity to a particular T cell cognate antigen.

The inhibition of immunosuppressive metabolites in the TME, has also demonstrated therapeutic potential. Gpr132 is a pH sensor expressed by macrophages and T cells. In a murine breast cancer model, inhibition of Gpr132 (and, thus, decreased sensitivity to lactate) increased M1 polarization while deterring the M2 phenotype, and this led to a shrinkage in tumor size (67). A ketogenic diet has been shown to reduce cancer cachexia by reducing systemic inflammation and limiting glucose and amino acid availability for cancer cells (54). About restoring DC function, a variety of measures have been proposed, including inhibition of FAO with etomoxir, an inhibitor of carnitine-palmitoyl transferase 1, the rate-limiting enzyme of this pathway; inhibition of mitochondrial fission with mdivi-1, rescuing the exhausted phenotype; and activating agonistic DC receptors, such as CD40, among others (56).

Turning our scope to parasitic infections, correcting metabolic abnormalities of immune cells could help combat these pathogens. Although there are no reports in the literature, some examples from protozoan parasites show this possibility. Effects of cerebral malaria in a murine model were reverted when an analog of glutamine was administered to mice. Purportedly, glutamine favored anabolism in exhausted and metabolically deranged macrophages and other immune cells, improving the response against the parasite (137). Likewise, supplementation of glutamine in the context of *Leishmania donovani* infection, to a base treatment of the antiparasitic agent miltefosine, increased its efficacy (138), likely by enhancing effector functions and “restoring” the glutamine pool lost to parasitism. This kind of interventions could prove useful in helminth infections. Zinc is essential in the orchestration of an effective Th2 response against infecting cestodes and nematodes, while selenium hones lymphocyte development and is also important in parasite expulsion through a Th2-mediated mechanism (139). Zinc supplementation, in addition, could prove beneficial in populations with endemic hypozincemia and/or at risk for parasitic infections (139).

Another attractive approach is the use of parasites to combat cancer. If parasites can alter immune plasticity through nutrient diversion and deprivation, as well as response skewing, it is certainly possible that similar phenomena could occur in a hypothetical parasite-tumor interface. Helminth-derived molecules from *F. hepatica* and *E. granulosus* could hold anti-tumor properties (140) as well as *Taenia solium* calreticulin (141). *E. granulosus* specifically can direct an immune response against certain tumor types and it, itself, be cytotoxic against tumor cells (142).

## 7 Conclusions

Immunometabolism, a relatively new discipline, has emerged as an approach to explain the intricate relationship between immune processes and metabolic pathways. This relationship, rather than being linear or unidirectional, is of the utmost complexity, and a change in a single level of interaction is capable of deeply affecting the dynamics between immunity and metabolism, between host and parasite, and between immune and cancer cells. As such, future therapeutic interventions could benefit from the simultaneous targets of the two axes, as elucidated in the above section. Further advances in cancer biology have allowed us to delve deeper into the mechanisms that allow malignant cells to avoid immune evasion, both individually and as part of a functional unit, i.e. the tumor. Both tumors and helminths induce regulatory Th2 responses and impact nutrient availability, which influences immune cell metabolism. However, they utilize different strategies and thus, immune cells exhibit distinct metabolic requirements and outcomes. Cancer cells create an acidic and hypoxic TME that modifies the anti-tumor immune responses. Both helminths and tumor cells can modify lipid metabolism and interact with the host adipose tissue. The changes promoted by cancer cells or helminths with their respective microenvironment, promote immune tolerance and evasion (**Table 3**). Given that tumor biology influences immune effector cell metabolism and vice versa, changes in either component, be it up-regulation of immune effector function or down-regulation of tumor metabolism, would likely have a positive effect on the other. A similar principle applies to parasites, whose capacity to drain the host of nutrients and re-program cells in its favor may, possibly, be abrogated by eliminating these alterations at a metabolic level. Certainly, the field of immunometabolism is ripe and ready to be explored.

**Table 3:** Comparative immune and metabolic changes in cancer and helminth infections.

	Cancer	Helminths
**Main immune alteration**	**Immunosuppression** **Immune evasion**	**Modified Th2 response** **Immune evasion**
Main immune cells involved	-Cytotoxic T lymphocytes (CD8^+^) -TAMs -T regs	-Th2 cells -M2 polarization -Tregs
Immunometabolic landmarks	-Nutrient deprivation (glucose and amino acids) -Warburg effect -Lactate production (generation of acidic TME) -Interaction with host lipid metabolism -Stimulation of FAO, OXPHOS in immune effector cells	-Nutrient deprivation (glucose and glutamine) -Interference with mTOR and other anabolic pathways -Interaction with host lipid metabolism -Stimulation of FAO, OXPHOS in immune effector cells
Net effects of immunometabolic alterations	-Immune tolerance against the tumor -Downregulation of immune surveillance -Tumoral growth, invasion and metastasis	-Immune modulation -Generation of an anti-inflammatory, tolerogenic microenvironment -Nutrient availability for parasite development and reproduction

## Author contributions

DE, AS-C, MI, MIMG, FM: wrote the manuscript; DE: Figure design and elaboration; AS-C, MIMG, DE: Table preparation; AS-C, MI, FM: conceptualization, design and reviewing of the manuscript. All authors contributed to the article and approved the submitted version.

## Glossary

**Table:**

Treg	regulatory T cells
Bregs	regulatory B cells
DCs	dendritic cells
TME	tumor microenvironment
ADCC	antibody-dependent cellular cytotoxicity
NK	natural killer
MHC	major histocompatibility complex
NCRs	natural cytotoxicity receptors
TAMs	tumor-associated macrophages
Arg1	arginase 1
TNF-α	tumor necrosis factor-α
CTLA-4	cytotoxic T-lymphocyte antigen-4
DAMPs	damage-associated molecular patterns
ILC2	type 2 innate lymphoid cells
E/S	excretory/secretory
TRM	tissue-resident memory T cells
FAO	fatty acid oxidation
FA	fatty acids
ETC	electron transport chain
OXPHOS	oxidative phosphorylation
TCR	T cell receptor
APC	antigen-presenting cell
PI3K	phosphatidyl-inositol 3 kinase
PKB/AKT	protein kinase B
mTOR	mammalian target of rapamycin
Teff	effector T cell
AMPK	adenosine monophosphate kinase
GLUT1	glucose transporter 1
LDH	lactate dehydrogenase
TCA	tricarboxylic acid
2-DG	2-deoxyglucose
iNOS	inducible nitric oxide synthase
NO	nitric oxide
TAGs	triacylglycerols
PPAR-γ	proliferating peroxisome activating receptor-γ
PGC-1β	PPAR-γ coactivator 1β
PRRs	pattern recognition receptors
TILs	tumor-infiltrating lymphocytes
MDSCs	myeloid-derived suppressor cells
HIF-1α	hypoxia-inducible factor 1α
MCTs	monocarboxylate transporters
LPS	lipopolysaccharide
PD-L1	programmed death-ligand 1
PD1	programmed cell death protein 1
H2O2	hydrogen peroxide
NSCLC	non-small cell lung cancer
CAFs	cancer-associated fibroblasts
mTORC1	mTOR complex 1
FhHDM-1	helminth defense molecule-1
LAL	lysosomal acid lipase
LDLs	low-density lipoproteins
VLDLs	very low-density lipoproteins
WAT	white adipose tissue
HOMA-IR	homeostatic model assessment for insulin resistance
T2DM	type two diabetes mellitus
